# A study on surface slant encoding in V1

**DOI:** 10.3389/fnsys.2013.00087

**Published:** 2013-11-15

**Authors:** Mohammed Sultan Mohiuddin Siddiqui, Basabi Bhaumik

**Affiliations:** Department of Electrical Engineering, Indian Institute of Technology DelhiNew Delhi, India

**Keywords:** visual cortex, surface slant selectivity, dif-frequency disparity, disparity selectivity, disparity map

## Abstract

Inter-ocular differences in spatial frequency occur during binocular viewing of a surface slanted in depth. Cortical cells with inter-ocular differences in preferred spatial frequency (dif-frequency cells) are expected to detect surfaces slanted in depth or vertical surface slant. Using our reaction-diffusion model, we obtain receptive fields and responses of simple cells in layer IV in cat V1. The dif-frequency cells in the model cortex have tilt in binocular receptive field but we show that tilt by itself does not indicate slant selectivity. We studied cell responses to binocular combination of spatial frequencies (SFs) by varying the SF ratio of the input gratings to the left and right eye in the range of 0.35–3. This range of SF ratio corresponds to surface slant variation of −85° to 85°. The mean binocular tuning hwhh (half width at half height) is 41°. Except for a small number (2.5%) of cells, most dif-frequency cells respond almost equally well for fronto-parallel surfaces. In the literature cells with inter-ocular difference in preferred orientation (IDPO) were expected to encode horizontal surface slant. In the model cat V1 mean hwhh in binocular orientation tuning curve for cells with IDPO is 39°. The wide binocular tuning width in dif-frequency cells and cells with IDPO imply that in cat V1 neither dif-frequency cells nor cells with IDPO detect surface slant.

## Introduction

The two eyes in humans and mammals are laterally separated and view this world from two different viewpoints. Binocular disparity is the difference between the left and the right retinal images. A cortical simple cell encodes binocular disparity of input stimuli for a small area of visual space (Hubel and Wiesel, 1962, 1968; Barlow et al., 1967; Nikara et al., 1968; Blakemore et al., 1972; Ferster, 1981; Ohzawa and Freeman, 1986a,b; LeVay and Voigt, 1988; Ohzawa et al., 1990, 1996, 1997; Anzai et al., 1999a,b) representing its receptive field (RF). Binocular disparity occurs mainly in three forms: (1) position disparity (Anzai et al., 1999a), (2) orientation disparity (Blakemore et al., 1972; Nelson et al., 1977; Bridge and Cumming, 2001), and (3) dif-frequency disparity (Tyler and Sutter, 1979; Sanada and Ohzawa, 2006).

Cortical simple cells encode position disparity through their left and right eye RFs' positional and phase disparities (Anzai et al., 1999a). Position disparity estimates depth of a fronto-parallel surface in visual space. Orientation disparity is the difference in orientation between the left and the right retinal images when viewing a surface slanted about horizontal axis so that the bottom edge of the surface is nearer than the top edge to an observer. Surface slant about the horizontal axis is referred as horizontal slant in this paper. Cortical neurons encode orientation disparity through IDPOs (Blakemore et al., 1972). Cells with IDPO are reported in cats (Blakemore et al., 1972; Nelson et al., 1977; Wieniawa-Narkiewicz et al., 1992) and monkeys (Bridge and Cumming, 2001). Using energy model Bridge et al. (2001) constructed complex cell RFs using simple cells made from Gabor filters and studied binocular tuning in cells with IDPO. Bridge et al.'s study qualitatively reproduced their electrophysiological results in monkey V1 and they reported that V1 cells with IDPO in monkey are not effective in horizontal slant detection.

Dif-frequency disparity is the difference in spatial frequency between the left and the right retinal images when an observer views a surface slanted about vertical axis. Surface slant about the vertical axis (referred as vertical slant in this paper) occurs when fronto-parallel surface is rotated away from the observer about its vertical axis so that the left edge is nearer than the right edge. Cortical neurons encode dif-frequency disparity through inter-ocular difference in their preferred SF. Dif-frequency cells are reported in cats (Hammond and Pomfrett, 1991) and in monkeys (Read and Cumming, 2003). But electrophysiological studies investigating binocular tuning in dif-frequency cells in V1 are not yet reported in the literature. Sanada and Ohzawa (2006) reported tilt in the binocular RF of dif-frequency selective cells in early visual areas 17 and 18 in cats. Tilt in RF indicates disparity gradient within RF. Sanada and Ohzawa therefore suggested that the encoding of 3D surface orientation, specifically encoding of vertical slant, begins in V1.

Recently we have proposed a reaction-diffusion model based on diffusive cooperation and resource limited competition for the development of left and right eye specific simple cell RFs (Siddiqui and Bhaumik, 2011). We had characterized disparity selective simple cells with matched preferred ORs and SFs in the left and the right eye, detecting fronto-parallel surfaces. In this paper we have studied response properties of cells with IDPO and dif-frequency using the reaction-diffusion model (Siddiqui and Bhaumik, 2011). In our model cortex, 43.16% (1079/2500) cells have IDPO and 55.2% (1380/2500) cells are dif-frequency selective. We have shown the following.

1. Dif-frequency cells are broadly tuned and therefore do not detect vertical slant. The tilts in binocular RF in the dif-frequency cells are similar to the ones reported in cats (Sanada and Ohzawa, 2006). However, tilt in binocular RF in itself, does not imply selectivity for vertical slant.
2. Cells with IDPO are broadly tuned and consequently are poor detector of horizontal slant. Our conclusion regarding cells with IDPO in model cat V1 is similar to Bridge and Cumming (2001)'s in monkey.

## Materials and methods

### A three layer visual pathway model

**Figure 1:**
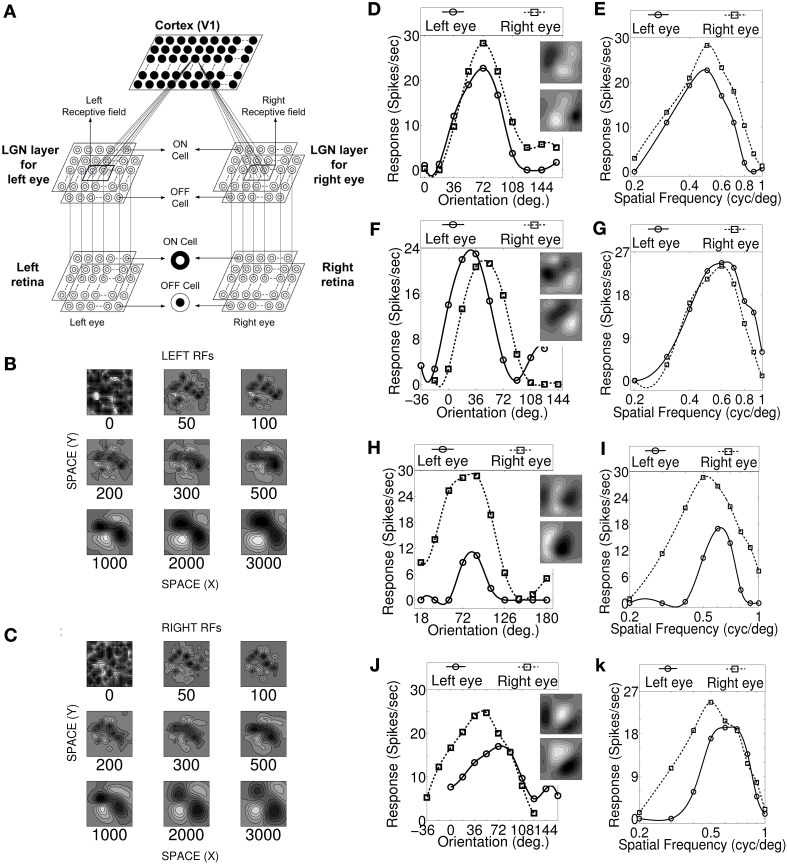
**Visual pathway model, RF development, OR and SF tuning curves. (A)** Three layer visual pathway model consists of (1) Layer 1: left and right retina/eye (each *M* × *M* overlapping ON and OFF retinal cells), (2) Layer 2: left and right eye specific LGN layers (each *M* × *M* overlapping ON and OFF LGN cells), and (3) Layer 3: layer IV of V1 in cat (*N* × *N* cortical cells). Each cortical cell in the model receives thalamic projections from 13 × 13 left and right eye specific LGN cells centered at their retinotopic center. These thalamocortical connections define left and right RFs. **(B,C)** The snap shots of the left and the right RF of a sample cell at different stages of development. The ON and OFF subregions are shown in gray scale with white (black) color representing strong synaptic connection from ON (OFF) LGN cells. The shading is proportional to the strength of the ON/OFF synaptic connections from LGN cells. **(D,F,H,J)** The left and the right monocular OR responses for four sample cells. The corresponding RFs are shown in the insets. **(E,G,I,K)** The left and the right monocular SF tuning responses for the same four sample cells. For characterization details please refer to Table 2. Sample cell 1 in **(D)** possess inter-ocular matched OR and matched SF preferences. Sample cell 2 in **(F)** possess inter-ocular unmatched OR (|IDPO| > 18°) and matched SF preferences. Sample cell 3 in **(H)** possess inter-ocular matched OR and unmatched SF (|dif-frequency| > 0.05 cycles/degree) preferences. Sample cell 4 in **(J)** possess inter-ocular unmatched OR and unmatched SF preferences.

In electrophysiological experiments sinusoidal gratings are shown to the left and the right eye to characterize the cortical cells. To characterize and compare our model cortical cells with the experimental results we have built a three-layer visual pathway consisting of the retina (left and right), the LGN (left and right eye specific), and the cortex. The three-layer visual pathway model is shown in Figure 1A (Siddiqui and Bhaumik, 2011). The first layer models left and right retinae. The retina for each eye is modeled as two separate 2D 30 × 30 sheets of ganglion cells lying one over the other. One sheet corresponds to ON center ganglion cells and the other to OFF center ganglion cells, respectively. We employ ganglion cell model that has been used earlier (Wehmeier et al., 1989; Wörgötter and Koch, 1991; Somers et al., 1995; Bhaumik and Mathur, 2003) to produce realistic temporal response to visual stimuli. The second layer models the left and the right eye specific LGN layers. Each LGN layer is also made up of two 2D 30 × 30 size sheets of LGN cells. One sheet comprised of ON center cells and the other of OFF center cells. It is reported that each LGN cell receives strong inputs from one to three retinal cells (Cheng and Regehr, 2000; Jaubert-Miazza et al., 2005). We assume that each LGN cell receives input from one retinal cell resulting in one-to-one connection between the retina (left and right) layers and the LGN (left and right eye specific) layers. Spike rates in the retina and the LGN are different (Carandini et al., 2007). We have incorporated this by changing the constant in Wörgötter and Koch (1991)'s retina model. We stimulate the model retina with a 50% contrast sinusoidal grating input and the spike rate in the model LGN cell is adjusted to match the experimental values (Cheng et al., 1995). The third layer models a 50 × 50 cortical layer IV of cat V1. Each cortical cell receives synaptic connections from 13 × 13 left and right eye specific ON/OFF LGN regions centered at its retinotopic position. The 13 × 13 left and right synaptic connections define the left and right RFs of a cortical cell. Thalamic projection of 13 × 13 LGN cells corresponds to inputs from approximately 4° × 4° visual space. We have used a modified (Bhaumik and Mathur, 2003) Spike Response Model (SRM) for obtaining cortical cell response (Gerstner, 1999). Details of the SRM model are given in (Bhaumik and Mathur, 2003).

We have used our thalamo-cortical synaptic weight development model (Bhaumik and Mathur, 2003; Siddiqui and Bhaumik, 2011), briefly summarized in the next subsection, to obtain the connections between the LGN and cortical cells. Biologically plausible competition and cooperation principles are used to model growth and decay of thalamo-cortical synaptic strengths. Both competition (reaction) and cooperation (diffusion) involves release of neurotrophic factors, neurotrophins which are activity dependent (Bonhoeffer, 1996; Cellerino and Maffei, 1996; Katz and Shatz, 1996; Lewin and Barde, 1996).

### Thalamo-cortical synaptic weight development: synaptic connection development from left and right specific LGN to cortex

In our model, *W*^*l*+^_*IJ*_ (*W*^*l*−^_*IJ*_) and *W*^*r*+^_*IJ*_ (*W*^*r*−^_*IJ*_), represents the strength of the connection from the ON (OFF) center LGN cell at position “*J*” in left and right eye specific LGN layer, respectively to the cortical cell at position “*I*” in the cortical layer. Synaptic connection development from the left eye specific ON center LGN to the cortex is governed by the equation given below:

(1) $$\frac{\partial W_{IJ}^{l+}}{\partial t}=(\gamma_{1}^{l}-K_{1}^{l})(\gamma_{2}-K_{2})A_{R}(I,\;J)C^{l+}A_{J}^{l+}W_{IJ}^{l+}+D_{L}\frac{\partial^{2}W_{IJ}^{l}}{\partial J^{2}}+D_{C}\frac{\partial^{2}W_{IJ}^{l}}{\partial I^{2}}$$

where, *W^l^_IJ_* ∈ {*W*^*l*+^_*IJ*_, *W*^*l*−^_*IJ*_}. The term (γ^*l*^_1_ − *K^l^*_1_) enforces competition for resources among axonal branches in a left eye specific ON center LGN cell. γ^*l*^_1_ is the total presynaptic resource available in the left LGN cell at location “*J*”. (*K^l^*_1_) represents the presynaptic resources already consumed at location “*J*”. ${\left(K_{1}^{l}\right)}^{2}=\sum_{P=1}^{N\times N}{\left(W_{PJ}^{l}\right)}^{2}$ is the sum of square of synaptic strength of all branches emanating from the LGN cell at the location “*J*”. *N* × *N* is the size of cortex layer. Similarly (γ_2_ − *K*_2_) enforces competition among LGN cells for target space in the cortex. γ_2_ is the total postsynaptic resource available at cortical cell at location “*I*”. (*K*_2_) represents the postsynaptic resources already consumed at that “*I*” location. ${\left(K_{2}\right)}^{2}=\sum_{P\;=\;1}^{M\;\times \;M}\left({\left(W_{IP}^{l}\right)}^{2}+{\left(W_{IP}^{r}\right)}^{2}\right)$ is the sum of square of synaptic strength of all branches of left and right eye LGN cells converging on the cortical cell at location “*I*”. *M* × *M* is the size of LGN layer. We have used *N* = 50 and *M* = 30. *A_R_*(*I*, *J*) is arbor function (Miller, 1994). The arbor function defines the region from where a cortical cell receives its initial unorganized thalamic afferents. The amount of afferents a cell receives is determined by the arbor window. A trapezoidal window (Miller, 1994), where the window height reduces as one move toward the periphery of the window, has been used for the results reported here.

Left and right eyes RFs of a cortical cell have subregions or subfields correspondence (Ohzawa et al., 1996) that leads to similar if not the same OR in the left and right eyes. While updating *W*^*l*+^_*IJ*_, subregions correspondence is achieved by taking

(2) $$C^{l+}=\{\begin{array}{ll} +1 & \text{if }W_{IJ}^{r}=W_{IJ}^{r+}\text{or }W_{IJ}^{r}=0\\ -1 & \text{if }W_{IJ}^{r}=W_{IJ}^{r-} \end{array}$$

For *C*^*l*+^ = +1, from LGN location “*J*” synaptic connections from both the left and the right eye are ON type. The active presynaptic input from the left and the right eye specific LGN cell at “*J*” add at the postsynaptic cell and *W*^*l*+^_*IJ*_ grows. For *C*^*l*+^ = −1, synaptic connection from the left eye is ON type but synaptic connection from the right eye is OFF type. Thus both the presynaptic inputs are not active at the same time and *W*^*l*+^_*IJ*_ decays. When we do not include *C*^*l*+^ in Equation (1), irrespective of whether the synaptic connections from the left and the right eye from LGN location “*J*” are both ON type or not, *W*^*l*+^_*IJ*_ grows and the left and the right eye RFs of a cortical cell do not have subregions or subfields correspondence.

*A*^*l*+^_*J*_ is the activity of ON center the left eye specific LGN cell at location “*J*”. While updating a synaptic weight between a cortical cell and an LGN cell, we assume that particular LGN cell to be active. For instance, while updating synaptic weight from the ON center LGN cell at position “*J*” in the left eye specific LGN, we put that LGN cell activity *A*^*l*+^_*J*_ = 1. *D*_*L*_ is the LGN diffusion constant. *D_C_* is the cortical diffusion constant.

Synaptic weight from the left eye specific OFF center LGN to the cortex is developed by updating using a differential equation obtained by replacing “l+” with “l−” in Equation (1). Similarly, synaptic connection development from the right eye specific LGN to the cortex *W*^*r*+^_*IJ*_(*W*^*r*−^_*IJ*_) is modeled by replacing “l” in the differential Equation (1) by “*r*.”

The influence of parameter variations, (1) LGN resource γ_1_, (2) cortical resource γ_2_, (3) LGN diffusion constant *D*_*L*_, and (4) cortical diffusion constant *D*_*C*_, are given in detail in Bhaumik and Mathur (2003). The value of LGN resource γ_1_ does not affect the structure of RF and the number of subregions (see Figure 6 in Bhaumik and Mathur, 2003). For low values of LGN resource γ_1_, synaptic weights between LGN cells and a cortical cell are quite weak due to scarcity of resources and as a result the cortical cell is not fully responsive to input stimuli. As the resources are increased the synaptic weights become stronger without affecting the number of sub regions and the structure of the RF.

LGN cells compete for cortical resource γ_2_. The number of simple cells with one subregion is greater for low values of γ_2_. LGN cells either ON-center type or OFF-center type take over the whole of the RF. The synaptic strengths for cortical cells with two or three subregions are too weak for γ_2_ ≤ 0.5 for the cortical cells to respond to input stimuli. The number of cells with two and three subfields increases with increase in γ_2_. For γ_2_ ≥ 1, with increase in cortical resources, the synaptic strengths increase, but the number of subfields remains the same (see Figure 5 in Bhaumik and Mathur, 2003).

The number of sub fields in the RF of a cortical cell increases as *D*_*L*_ is reduced (see Figure 9 in Bhaumik and Mathur, 2003). For results presented in this paper we have taken *D*_*L*_ = 0.0125. We have also developed RFs with eight different values of *D*_*L*_ by setting *D*_*L*_ = 0.0125X and varying *X* from 0.125 to 2.0. With *X* = 0.125 i.e., *D*_*L*_ = 0.0015625, RFs of most cells have a large number of sub-fields ranging from four to six. On the other hand with *X* = 2.0, i.e., *D*_*L*_ = 0.05, most cells have a single sub-region in their RFs. For 0.75 ≤ *X* ≤ 1.25, we get RFs having one, two, or three sub-regions in the model cortex as reported in the literature. Most cells have two sub-regions in their RFs.

*D*_*C*_ ensures that near neighbor cells have similar RFs and OR preferences (see Figure 8 in Bhaumik and Mathur, 2003) as reported in DeAngelis et al. (1999). We have also developed RFs using different seeds for initial random weight distribution. The RFs developed and the cell response characteristics obtained for different seeds are qualitatively similar and show similar distribution of preferred binocular phase disparity distribution. Therefore, the result presented in this paper is robust. We use LGN diffusion constant, *D*_*L*_ = 0.0125, cortical diffusion constant, *D*_*C*_ = 0.0075, LGN resources, γ^*l*^_1_ = γ^*r*^_1_ = 1, and cortical resource, γ_2_ = 1.5. A list of variables and parameters along with their description is provided in Table 1.

**Table 1:** **List of variables and model parameters**.

*W*^*l*+^_*IJ*_	Synaptic weight from ON center LGN cell at position “*J*” in the left eye specific LGN layer to a cortical cell at position “*I*” in the model cortex
*W*^*l*−^_*IJ*_	Synaptic weight from OFF center LGN cell at position “*J*” in the left eye specific LGN layer to a cortical cell at position “*I*” in the model cortex
*W*^*r*+^_*IJ*_	Synaptic weight from ON center LGN cell at position “*J*” in the right eye specific LGN layer to a cortical cell at position “*I*” in the model cortex
*W*^*r*−^_*IJ*_	Synaptic weight from OFF center LGN cell at position “*J*” in the right eye specific LGN layer to a cortical cell at position “*I*” in the model cortex
*A_R_*(*I*, *J*)	Arbor function
*A*^*l*+^*_J_*	Activity of ON center LGN cell at position “*J*” in the left eye specific LGN layer
*C*^*l*+^	Subregion correspondence factor
γ^*l*^_1_	Presynaptic resource available in the left LGN cell
γ^*r*^_1_	Presynaptic resource available in the right LGN cell
γ_2_	Postsynaptic resource available in the cortical cell
*D_L_*	LGN diffusion constant
*D_C_*	Cortical diffusion constant

Development of the left and the right RF structures of a sample cortical cell at different stages of development are shown in Figures 1B,C, respectively. The ON and the OFF subregions are shown in gray-scale with white (black) color representing strong synaptic connection from ON (OFF) LGN cells. The shading is proportional to the strength of the ON/OFF synaptic connections from LGN cells. At epoch 0, ON and OFF synaptic connections from the left (*W*^*l*+^_*IJ*_, *W*^*l*−^_*IJ*_) and the right (*W*^*r*+^_*IJ*_, *W*^*r*−^_*IJ*_) eye LGN cells, forming left and right RFs, respectively, are randomly organized. At around epoch 100, the left and the right RFs of the cortical cell develop small patches of ON or OFF subregions. The formations of patches occur due to cooperation among ON (OFF) synapses helping other neighboring ON (OFF) synapses to grow and push out any OFF (ON) synapses existing in a patch. The cooperation phenomenon is gradual and is due to diffusion in the LGN. At epoch 3000, RFs have well defined segregated ON and OFF subregions with gradual transition from ON (OFF) subregion to OFF (ON) subregions. To the best of our knowledge, reaction diffusion model (Bhaumik and Mathur, 2003; Siddiqui and Bhaumik, 2011) captures not only the most realistic looking simple cell RFs but also captures the single cell, cell population and map properties reported by experimentalists.

### Determination of or and SF preferences, OD, and DP

We stimulated the model retina with sinusoidal grating and obtained cortical cell's spike response. The sinusoidal gratings are of 50% contrast at 0.5 cycles/degree spatial frequency and moving at a velocity of 2 degrees/s. The direction of motion of the grating is always orthogonal to the orientation of the grating.

Left monocular OR preference of the cortical cells are obtained by stimulating the left retina with sinusoidal gratings of different ORs varying from 0° to 180° in steps of 18°, and the right retina with zero input. Each orientation was presented to the retina thirty times. Spike rates per second were computed for individual bins of 100 ms width each and the response was then averaged over the thirty-recorded Peristimulus time histograms. The cell spike response for any given orientation of input stimulus is the maximum response obtained in the averaged histogram. Ten responses were obtained for ten orientations of input stimulus. A cubic spline curve is fitted through these ten responses of the cortical cell to obtain OR tuning curve. The preferred left monocular OR is the OR at which the cell responds most vigorously. The right monocular orientation preference of the cortical cell is obtained by stimulating the right retina and the binocular orientation preference by stimulating both the retinae.

We have also obtained the left and the right monocular OR tuning curves for a number of sample cells stimulating the retina with sinusoidal gratings of different ORs varying from 0° to 180° at finer steps of 6° and at steps of 18°. The orientation preference and hwhh remain unaffected. So, to save computational time, we varied input grating stimulus OR at 18° steps.

We measure SF tuning of each cortical cell by stimulating our modeled retina by sinusoidal grating with spatial frequencies varying from 0.1 to 1 cycles/degree in steps of 0.1 cycles/degree with OR of the grating fixed at the cell's preferred OR. A cubic spline curve is then fitted to these responses to obtain their SF tuning curve. The optimal SF is the spatial frequency at which the cell responds most vigorously.

OD is computed using the expression given in Albus (1975). OD = (*R*_*r*_ − *R*_*l*_)/(*R*_*r*_ + *R*_*l*_), where *R*_*r*_ (*R*_*l*_) is the sum of the right (left) monocular responses of cortical cell to sinusoidal grating.

Binocular phase disparity tuning for a cell is obtained by dichoptically stimulating modeled retinae with drifting sinusoidal gratings at cell's preferred OR. The relative phase difference (also referred as relative phase disparity) between the dichoptically shown sinusoidal gratings were varied from 0° to 360° in steps of 18° and the cortical cell response was obtained. The response data is fitted with a cycle of sine wave using least square criterion to obtain a disparity tuning curve. The relative disparity phase at which the fitted sine wave peaks gives the preferred binocular phase disparity (DP). The ratio of the amplitude of a sine-fitted disparity tuning curve to its mean response amplitude is defined as binocular interaction index (BII) (Ohzawa and Freeman, 1986a; Smith et al., 1997).

## Results

### Response characterization

In our model 50 × 50 cortex, 1732 cells out of total 2500 cells i.e., 69.3% of cells are OR tuned. The rest 30.7% cells are OR untuned in at least one eye. OR preference of the untuned cells is obtained using vector addition method (Blasdel, 1992). Cells in the model cortex developed with *D*_*L*_ = 0.0125, have SF in the range of 0.2–0.85 cycles/degree. We can achieve a wider SF range of 0.19–1.04 cycles/degree by varying *D*_*L*_ parameter in our simulation (Mathur and Bhaumik, 2005). Experimental finding in cats reports SF range of 0.3–1.8 cycles/degree (Andrews and Pollen, 1979). Our simulated cortical cells SF range lacks in covering high SFs as compared to the experimentally observed SF range in cats. We attribute this difference to fixed center size (30') retinal X-cell used in our model retinae (Siddiqui and Bhaumik, 2011). In cats, retinal X-cell center size varies from 20' in the central area to about 40' at an eccentricity of 0.75 mm (see Figure 7 in Peichl and Wässle, 1979). Broader range of SF can be achieved by incorporating retinal X-cells with different center sizes in our model.

**Figure 2:**
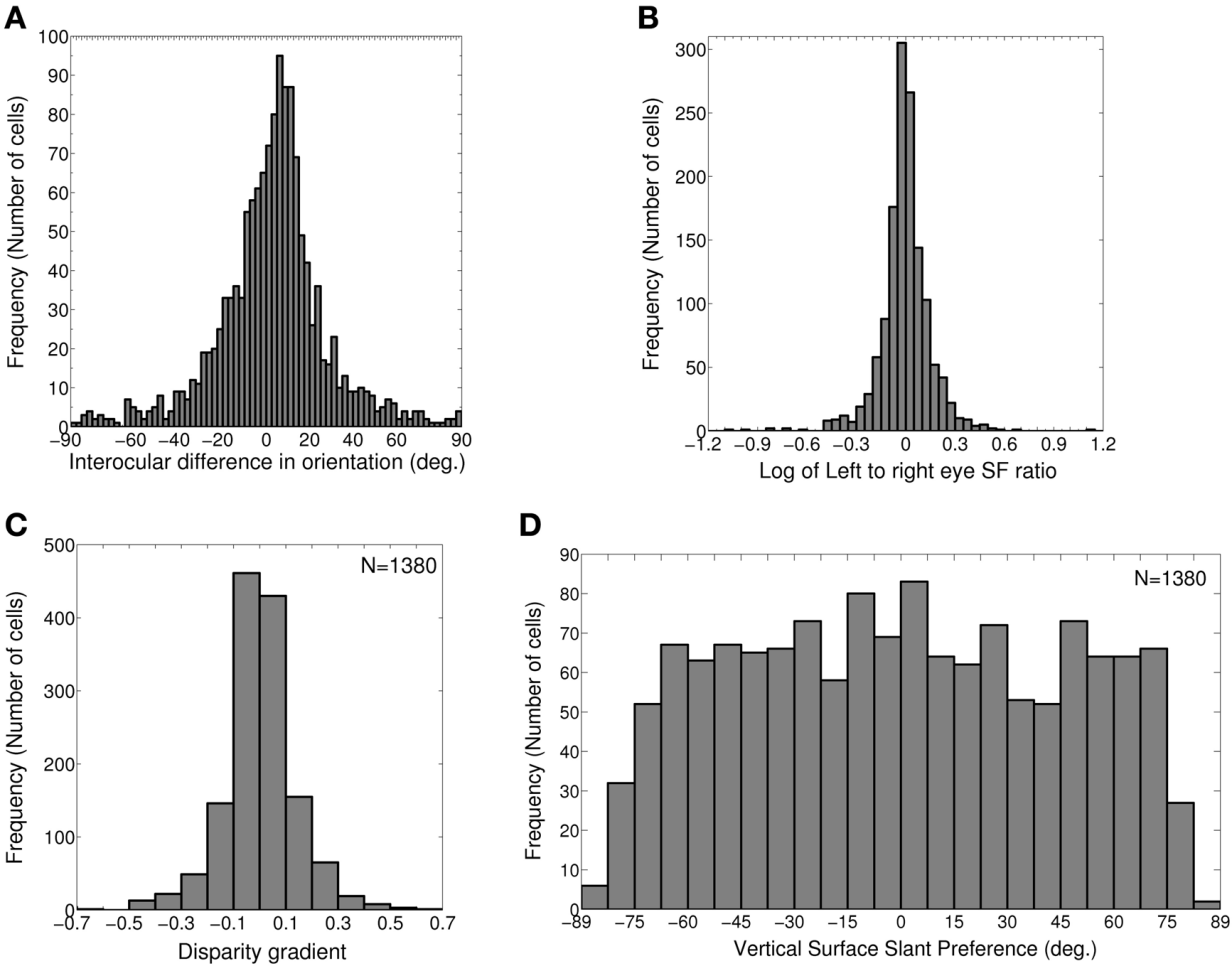
**IDPO, left to right eye SF ratio, Disparity gradient and Vertical surface slant preference. (A)** Histogram of IDPOs in degrees. 73.4% of cells have IDPOs in the range of ±20° (*S* = 8.9°). Rest of the cells has significant IDPOs (|IDPO| > 20°). **(B)** Histogram of log of monocular left to right eye SF ratio for 1380 modeled cells with matched OR preferences in the two eyes. 86.4% cells have monocular left to right eye SF ratio in the range of 0.8–1.2. 5.4% cells have monocular SF ratio <0.8 and 8.3% cells have monocular SF ratio >1.2. **(C)** Histogram of disparity gradient of the cells in **(B)**. The disparity gradient lies in the range ±0.95 (*S* = 0.15). **(D)** Histogram of vertical surface slant preference for cells in **(B)**.

**Figure 3:**
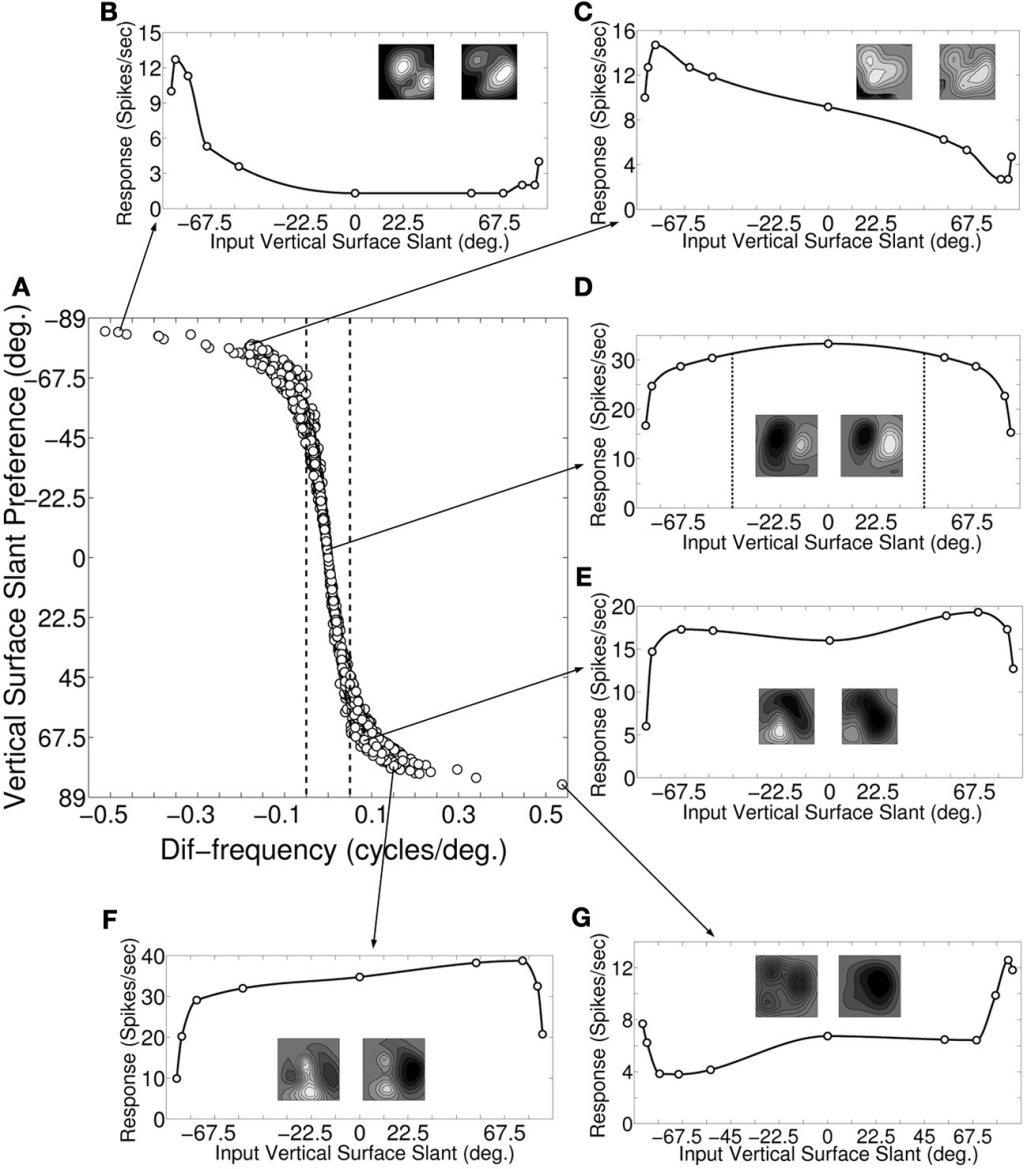
**Slant preference and response of vertical slant selective cells. (A)** Scatter plot of dif-frequency versus vertical surface slant selectivity for our modeled cortical cells (*N* = 1380). The dotted demarcation lines represent |dif-frequency | = 0.05 cycles/degree. **(B–G)** Depicts vertical surface slant tuning for six sample cells along with their monocular receptive fields. The arrow lines indicate where these sample cells are located in the scatter plot **(A)**.

**Figure 4:**
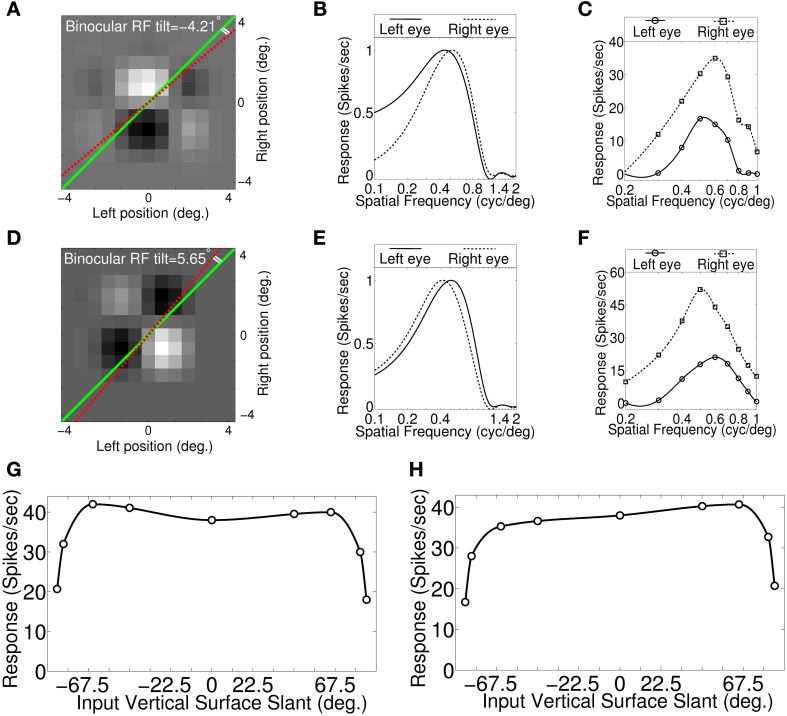
**Binocular RF, monocular spatial frequency tuning curves and Vertical surface slant tuning curves. (A)** Binocular RF of a sample cell from our modeled cortex. The dotted red line and the green line indicate RF tilt angle and fronto-parallel line, respectively. This cell possesses significant RF tilt of −4.21°. **(B)** Estimated monocular left and right eye spatial frequency tuning curves from binocular RF spectral profile. **(C)** Monocular left and right spatial frequency tuning curves obtained by stimulating model retina with sinusoidal grating. Monocular preferred spatial frequency ratio is 0.87. **(D)** Binocular RF of another sample cell. RF tilt angle for this cell is 5.65°. **(E)** Estimated monocular left and right eye spatial frequency tuning curves from binocular RF spectral profile. **(F)** Monocular left and right spatial frequency tuning curves obtained by stimulating retina with sinusoidal grating. Spatial frequency ratio is 1.2. **(G)** Vertical surface slant tuning curve for the cell shown in **(A)**. **(H)** Vertical surface slant tuning curve for the cell shown in **(D)**.

We characterized our model cortical cells by ascertaining their monocular (left/right) OR preference, monocular SF preference and OD (see the section Materials and Methods). Cells with |IDPO| ≤ 18° are classified as cells having matched OR preferences in the left and the right eye. Cells with |dif-frequency| ≤ 0.05 cycles/degree are classified as cells with matched SF preferences in the two eyes. The modeled cortical cells form four groups having: (1) matched OR and matched SF preferences (see Figures 1D,E), (2) unmatched OR (|IDPO| > 18°) and matched SF preferences (see Figures 1F,G), (3) matched OR and unmatched SF (|dif-frequency| > 0.05 cycles/degree) preferences (see Figures 1H,I), and (4) unmatched OR and unmatched SF preferences (see Figures 1J,K).

We present the left and the right monocular OR tuning curves and SF tuning curves of four sample cells, each belonging to one of these four groups along with their RFs in Figures 1D–K. The details regarding OR and SF preferences in the left and the right eyes, IDPO, dif-frequency, and OD for the four sample cells are listed in Table 2. The ON and OFF regions in RFs (see Figures 1D,F,H,J) are shown in gray-scale with white (black) color representing strong synaptic connection from ON (OFF) LGN cells. The shading is proportional to the strength of the ON/OFF synaptic connections from LGN cells.

**Table 2:** **OR and SF preferences in left and right eyes, IDPO, dif-frequency, and OD for four sample cells**.

**Sample cells**	**Preferred OR (degree)**		**IDPO (degree)**	**Preferred SF (cycles/degree)**		**Dif-frequency (cycles/degree)**	**OD**
	**Left eye**	**Right eye**		**Left eye**	**Right eye**	
Cell 1	72	72	0	0.48	0.51	−0.03	0.16
Cell 2	28	48	20	0.64	0.61	0.03	−0.09
Cell 3	83	83	0	0.62	0.52	0.1	0.73
Cell 4	77	47	30	0.65	0.5	0.15	0.16

In our model cortex, 1079 out of 2500 (43.16%) cells belong to groups (1) and (2). These cells have the same preferred spatial frequency in both the eyes. We find the IDPO for these cells, and plot the histogram for IDPO with bin width of 2.5° in Figure 2A. 73.4% OR tuned cells (792 out of total 1079) have IDPO in the range of ±20° (*S* = 8.9°). Rest of the cells have significant IDPOs (|IDPO| > 20°).

1380 out of 2500 (55.2%) cells in the model cortex belong to groups (1) and (3). These cells have a matched OR preference in the left and the right eye. We determine monocular left to right eye preferred SF ratio for these cells, and plot the histogram of preferred SF ratio in Figure 2B. 86.4% cells (1192 out of total 1380) have monocular left to right eye preferred SF ratio in the range of 0.8–1.2. 5.4% cells (74 out of total 1380) have monocular preferred SF ratio <0.8. 8.3% cells (114 out of total 1380) have monocular preferred SF ratio >1.2.

### Disparity gradient and slant from monocular responses

Cells with dif-frequency are expected to detect vertical surface slant. Vertical surface slants of 3D oriented surfaces are quantified by disparity gradient. Disparity gradient is a function of preferred SF ratio in the left and the right eye (Sanada and Ohzawa, 2006). The equation for disparity gradient (Δ*d*) is as follows:

(3) $$\Delta d=2\left(\frac{f_{\text{ratio}}-1}{f_{\text{ratio}}+1}\right)$$

For faithful binocular fusion, the absolute value of disparity gradient for two dots must be < 1 − 2 depending on the exact dot parameters (Burt and Julesz, 1980; Prazdny, 1985; Trivedi and Lloyd, 1985). We would expect our model cortical cells to encode disparity gradient within this limit. We have determined disparity gradient for our model cortical cells. Figure 2C depicts histogram of disparity gradient for our model cortex. The disparity gradient lies in the range ±0.95 (*S* = 0.15). However, most of the neurons had disparity gradients within a much tighter range of ±0.5 (see Figure 2C). This is similar to the results reported in Figure 9 in Sanada and Ohzawa (2006). Disparity gradient range for our cells is well within the binocular fusion range. This range of disparity gradient is capable of representing surface slants in the range ±85° from the fronto-parallel plane at 50 cm fixation distance in real 2D visual space (Tyler and Sutter, 1979; Sanada and Ohzawa, 2006). Next, we ascertained whether these cells show any OR bias for disparity gradient or not. To check this, we obtained correlation between disparity gradient and binocular OR preferences. We obtained no correlation (*r* = 0.02). This conforms to experimental findings by Sanada and Ohzawa (2006).

From the monocular spatial frequency preferences of the two eyes we have also obtained vertical slant preference (ϕ_υ_) for dif-frequency cells. ϕ_υ_ is calculated using the equation (Tyler and Sutter, 1979; Sanada and Ohzawa, 2006):

(4) $$\text{tan}(\phi_{\upsilon })=\left(\frac{f_{\text{ratio}}-1}{f_{\text{ratio}}+1}\right)cot(\upsilon /2)$$

where, *f*_ratio_ = *f_l_*/*f_r_*, monocular left to right eye preferred SF ratio, υ is the vergence angle. The inter-pupillary distance between the two eyes (2a), and fixation distance (d) determine the vergence angle [υ = 2 tan^−1^(*a*/*d*)]. The inter-pupillary distance (2a) between the two eyes of cats is 4.2 cm. In experimental studies, a fixation distance of 50 cm is often used for cats and macaques. So, we choose a fixation distance (d) to be 50 cm and obtain the preferred surface slants for our model cortical cells. Figure 2D depicts the histogram of vertical surface slant preference, ϕ_υ_ for dif-frequency cells. ϕ_υ_ is almost uniformly distributed in the range of ± 75°. A small number of cells have ϕ_υ_≤ −75° or ≥ 75°. Overall vertical surface slant preference lies in the range of ±85° (*S* = 48.8°).

The disparity gradient and vertical slant preference (ϕ_υ_) discussed above are obtained from monocular spatial frequency response and do not necessarily indicate slant detection sensitivity or ability of dif-frequency selective cell. To determine the slant tuning response we next study the binocular responses of dif-frequency cells in our model cortex.

### Binocular responses: encoding vertical slant

Figure 3A depicts a scatter plot of vertical surface slant preference (ϕ_υ_) calculated from monocular response versus dif-frequency for model cortical cells. The dotted demarcation lines represent |dif-frequency| = 0.05 cycles/degree. The difference in the preferred spatial frequency between the two eyes ranges from small values to substantially large values of over an octave. We next choose 50 sample cells from Figure 3A and obtain binocular response. To obtain binocular response we have stimulated the left and the right retina with different combination of SFs in the left and the right eye. SF ratio of the input gratings to the left and the right eye was varied in the range of 0.35–3. For each value of the SF ratio we calculate the corresponding input surface slant (ϕ^*in*^_υ_) value using Equation (4). SF ratio variation in the range of 0.35–3 corresponds to variation of ϕ^*in*^_υ_ from −85° to 85°. We have also quantified the slant tuning strength through the maximum (max) and the minimum (min) cortical cell response in the slant tuning curve. Vertical slant tuning strength is defined as (max − min)/(max) (Hinkle and Connor, 2002). Figures 3B–G depict binocular response for six sample cells as a function of input surface slant (ϕ^*in*^_υ_). The arrow lines indicate where these sample cells are located in the scatter plot of Figure 3A. The monocular receptive fields of the six sample cells are shown along with their slant tuning responses. The six sample cells belong to the three categories with (1) dif-frequency = 0 cycles/degree, (2) dif-frequency <−0.05 cycles/degree, and (3) dif-frequency >0.05 cycles/degree, respectively.

Figure 3D shows the slant tuning plot for a sample cell with dif-frequency = 0 cycles/degree. The left and the right monocular preferred SFs for the cell are 0.5 and 0.5 cycles/degree, respectively i.e., ϕ_υ_ = 0°. The slant tuning strength for this cell is 0.54. It is evident from the slant tuning characteristics that such a cell responds very well (within 7% of maximum response) for input surface slant, ϕ^*in*^_υ_ in the range of −45° to 45°. For detecting large extended surfaces it is expected that inputs are pooled from V1. The flat tuning curve in Figure 3D is interesting, given that human observers are very bad at detecting horizontal disparity gradients (Rogers and Graham, 1983) for large extended surfaces.

Figures 3B,C show the slant tuning plots of two sample cells with dif-frequency < −0.05 cycles/degree. The slant tuning plot shown in Figure 3B shows sharp tuning with slant tuning strength of 0.9. For this cell, the left and right monocular preferred SFs are 0.3 and 0.7, respectively and ϕ_υ_ = −84°. The right monocular preferred SF is more than 1 octave away with respect to the left monocular preferred SF. Figure 3C depicts response of the other sample cell. For this cell ϕ_υ_ = −81° with the left and right monocular preferred SFs being 0.4 and 0.7, respectively. The slant tuning strength is 0.82. This cell also has relatively sharp tuning characteristics.

Figures 3E–G show the slant tuning response of three sample cells with dif-frequency >0.05 cycles/degree. The cell for Figure 3E has the left and the right monocular preferred SFs of 0.5 and 0.4 cycles/degree, respectively i.e., ϕ_υ_ = 69°. The slant tuning strength for this cell is 0.69. The cell has wide tuning width. Similarly, the response of the cell shown Figure 3F also has a wide vertical slant tuning width. For this cell, the left and right monocular preferred SFs are 0.7 and 0.5 cycles/degree, respectively i.e., ϕ_υ_ = 75.8°. The slant tuning strength is 0.74. For the cell response shown in Figure 3G, ϕ_υ_ = 84° with the left and right monocular preferred SFs being 0.7 and 0.3 cycles/degree, respectively. The left monocular preferred SF is more than one octave away with respect to the right monocular preferred SF. The slant tuning strength is 0.7. The cell has sharp slant tuning. Slant tuning strength is not a good indicator of how narrow or flat tuning width is. For instance, two cells whose responses depicted in Figures 3F,G have similar slant tuning strength, but their tuning widths are vastly different. We found no correlation (*r* = −0.19) between slant tuning strength and preferred slant angle.

Most of the dif-frequency cells in our model cortex show poor vertical slant tuning characteristics and consequently will not encode vertical slant effectively. Only small number of dif-frequency cells [2.5% (35/1380)] with monocular SF ratio either ≤ 0.67 (ϕ_υ_≤ −78°) or ≥ 1.49 (ϕ_υ_ ≥ 78°) have sharp slant tuning characteristics similar to vertical slant tuning characteristics reported in V4 by Hinkle and Connor (2002). These cells also possess strong slant tuning strengths (≥ 0.9).

We next determine the tilt in the binocular interaction RF of dif-frequency cells to ascertain whether tilt in binocular RF indicates slant selectivity.

### Tilt in binocular RF

Sanada and Ohzawa (2006) had mapped binocular interaction RF of dif-frequency selective cortical cells using reverse correlation method and reported existence of tilt in the binocular RF of dif-frequency cells. The tilt in binocular interaction RF indicates that the preferred disparity of the cell is changing within the RF. We obtained binocular interaction RFs of our model cortical simple cells to ascertain RF tilts for 316 sample cells. Separable type binocular interaction RFs are reported for simple cells (Anzai et al., 1999b). We, therefore, obtained binocular RFs by multiplying left and right eye 1D RF profiles of our model cortical simple cells. We computed binocular RF tilts for the sample cells using the method given in Sanada and Ohzawa (2006). We calculated disparity gradient (Δ*d*) from binocular RF tilt angle (θ) using the formula (Sanada and Ohzawa, 2006):

(5) $$\Delta d=2\left(\frac{1-\tan(45-\theta )}{1+\tan(45-\theta )}\right)$$

Figures 4A,D show binocular RF of the two sample cells from the model cortex. The two sample cells have wide slant tuning width (see Figures 4G,H). In Figures 4A,D the dotted red line, and the green line, respectively indicate RF tilt line and the fronto-parallel axis. The cell in Figure 4A possesses significant RF tilt of −4.21° and the corresponding disparity gradient using Equation (5) is 0.147. Two sets of monocular left and right eye spatial frequency tuning curves are shown in Figures 4B,C. Tuning curves shown in Figure 4B are obtained from binocular RF spectral profile. Tuning curves shown in Figure 4C are obtained by stimulating the model retina with sinusoidal grating. The monocular spatial frequency ratio is 0.87 (see Figure 4C) and the corresponding disparity gradient using Equation (3) is −0.14. The disparity gradient obtained using binocular RF tilt and spatial frequency ratios from monocular responses are quite similar.

The cell in Figure 4D also possesses significant RF tilt of 5.65°. The corresponding disparity gradient for the cell is 0.2. The monocular left and right eye spatial frequency tuning curves obtained from binocular RF spectral profile, and by stimulating the retina with sinusoidal grating are shown, respectively in Figures 4E,F. Preferred spatial frequency ratio obtained from the monocular response is 1.2 and the disparity gradient for the cell is 0.18. The RF tilt values of the two samples cells shown in Figures 4A,D are similar to the experimentally reported tilts in simple cell RFs (see Figures 6B,C in Sanada and Ohzawa, 2006). The tilt value by itself in the two sample cells would suggest that these two cells would detect slant. But the slant tuning characteristic of the two cells are almost flat over −67.5° to 67.5° (see Figures 4G,H). This illustrates that the tilt in the binocular RF does not indicate a cell's ability to encode vertical slant.

We have obtained disparity gradient for the sample cells (*N* = 316) from binocular RF tilts in these cells. The disparity gradient lies in the range ±0.5. This range agrees with experimentally reported value (Burt and Julesz, 1980; Prazdny, 1985; Trivedi and Lloyd, 1985). We obtained slight positive correlation (*r* = 0.39, *P* < 0.01, Spearmans correlation coefficient) between disparity gradient obtained using RF tilt and SF frequency ratio for our cells. A lower correlation (*r* = 0.27, *P* > 0.05) coefficient is reported for simple cells in V1 (Sanada and Ohzawa, 2006). The number of samples (*N* = 14) in Sanada and Ohzawa (2006) is quite small.

In this and the preceding sections we have shown that dif-frequency cells do not detect vertical slant. We next study the binocular responses of cells with IDPO in our model cat V1.

### IDPO and encoding horizontal slant

**Figure 5:**
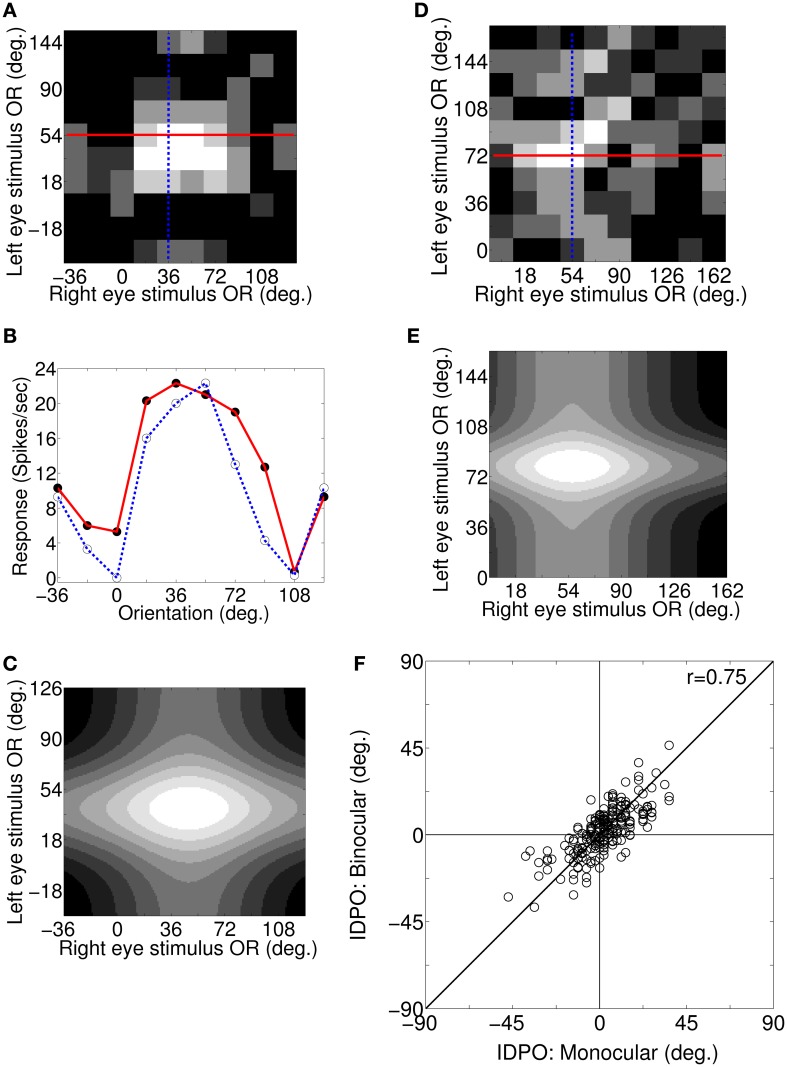
**Binocular responses: surface plot, 1D plot, 2D Gaussian fit, and monocular IDPO versus binocular IDPO. (A)** Binocular response surface plot for a sample cortical cell. Columns and rows represent OR of the grating applied to left and right eye, respectively. Binocular firing rate for each pair of input stimulus to the left and the right eye is represented in gray-scale. Low firing rates is represented by darker shades and the high firing rates by lighter shades. At the crossing of the solid red line and the blue dotted line in **(A)**, OR of grating to the left and right are 54° and 36°, respectively and the binocular response of the cell is 22 spikes/s. **(B)** Two set of 1D binocular response for the same cell. In the first set (red curve), OR for the grating to the left eye is fixed at the cell's preferred OR of 54° and OR of the grating applied to the right eye is varied. In the second set (blue dotted curve), OR of the grating to the right eye is fixed at the cell's preferred OR of 36° and OR of the applied grating to the left eye is varied. **(C)** Fitted 2D Gaussian function to the binocular response surface plot for the sample cell in **(A)**. The peak of the fitted 2D Gaussian function center gives the cell's binocular IDPO value. The cell's binocular IDPO is 10°. **(D)** Binocular response surface plot for another sample cortical cell. The maximum binocular response of this cell is 29 spikes/s. **(E)** Fitted 2D Gaussian function to binocular response surface plot for the sample cell in **(D)**. The cell's binocular IDPO is 22°. **(F)** Scatter plot of monocular IDPO versus binocular IDPO for 210 modeled simple cells. The monocular IDPO and binocular IDPO show strong correlation between them (*r* = 0.75, *N* = 210).

For a cortical cell with IDPO to be effective in detecting surface slant about horizontal axis the cell must have sufficient binocular interaction. We obtain the response of cells to different binocular combination of grating orientations (Bridge and Cumming, 2001) applied to the two eyes. Figure 5A depicts the binocular response surface plot for a sample cortical cell. The columns and the rows represent OR of the grating applied to the left the and right eye, respectively. Binocular firing rate for each pair of input stimulus to the left and the right eye is represented in Gray-scale. Low firing rate is represented by darker shades and the high firing rate by lighter shades. At the crossing of solid red line and blue dotted line in Figure 5A, OR of grating to the left and the right eye are 54° and 36°, respectively and the binocular response of the cell is 22 spikes/s. Figure 5B depicts two sets of 1D binocular response for the same cell. In the first set (red curve), OR of grating to left eye is fixed at the cell's preferred OR of 54° and OR of the grating applied to the right eye is varied. In the second set (blue dotted curve), OR of grating to the right eye is fixed at the cell's preferred OR of 36° and OR of the applied grating to the left eye is varied. In Figure 5B, binocular response (depicted with red solid line) is almost similar when input gratings ORs to right eye are at 18°, 36°, 54°, and 72°, showing almost flat tuning response. Similarly, binocular response (depicted by blue dotted line) is almost similar when left eye is stimulated with grating having ORs 36° and 54°. These wide OR tuning widths in binocular response suggest no specialization for signaling horizontal surface slant in the cells with IDPO.

We have fitted a 2D Gaussian function to binocular response surface plot to compute cell's binocular IDPO value. IDPO value, at which the fitted Gaussian peaks, is the binocular IDPO. Figure 5C depicts the 2D fitted Gaussian function to the binocular response surface plot of Figure 5A. The cell's binocular IDPO is 10°. Figure 5D depicts binocular response surface plot for another sample cell from our model cortex. At the crossing of the solid red line and the blue dotted line in Figure 5D, the OR of the stimulus for left and right eye are 72° and 54°, respectively and the binocular response of the cell is 29 spikes/s. The IDPO obtained from monocular response is referred to as monocular IDPO. Monocular IDPO for this cell is 18°. The tuning width of this cell is also quite wide. We have fitted 2D Gaussian function to this cell's binocular response surface plot. The fitted Gaussian is shown in Figure 5E. The binocular IDPO value is 22°. We obtained binocular IDPO for 210 cells by fitting 2D Gaussian functions. Figure 5F depicts scatter plot of monocular IDPO versus binocular IDPO. The monocular IDPO and binocular IDPO show strong correlation between them (*r* = 0.75, *N* = 210).

Bridge and Cumming (2001) had reported that cells with IDPO in monkeys have broad binocular tuning response and do not detect horizontal slant. In our model cortex for cat V1 we too have found that cells with IDPO have broad binocular tuning width. Bridge and Cumming (2001) had reported no significant correlation (*r* = 0.26, *N* = 45) between binocular IDPO and monocular IDPO. The samples chosen by Bridge and Cumming are mostly complex cells in monkey V1, whereas our modeled cells are simple cells in a model cat V1.

Dif-frequency cells as well as cells with IDPO possess spatial offsets between left and right eye RFs. RF spatial offset between the left and the right eyes endows cortical cells with disparity selective property. We therefore, expect that these cells will encode binocular preferred phase disparity (DP).

### Binocular preferred phase disparity

We have determined binocular preferred phase disparity (DP) of dif-frequency cells, and cells with IDPO.

#### Dif-frequency cells

**Figure 6:**
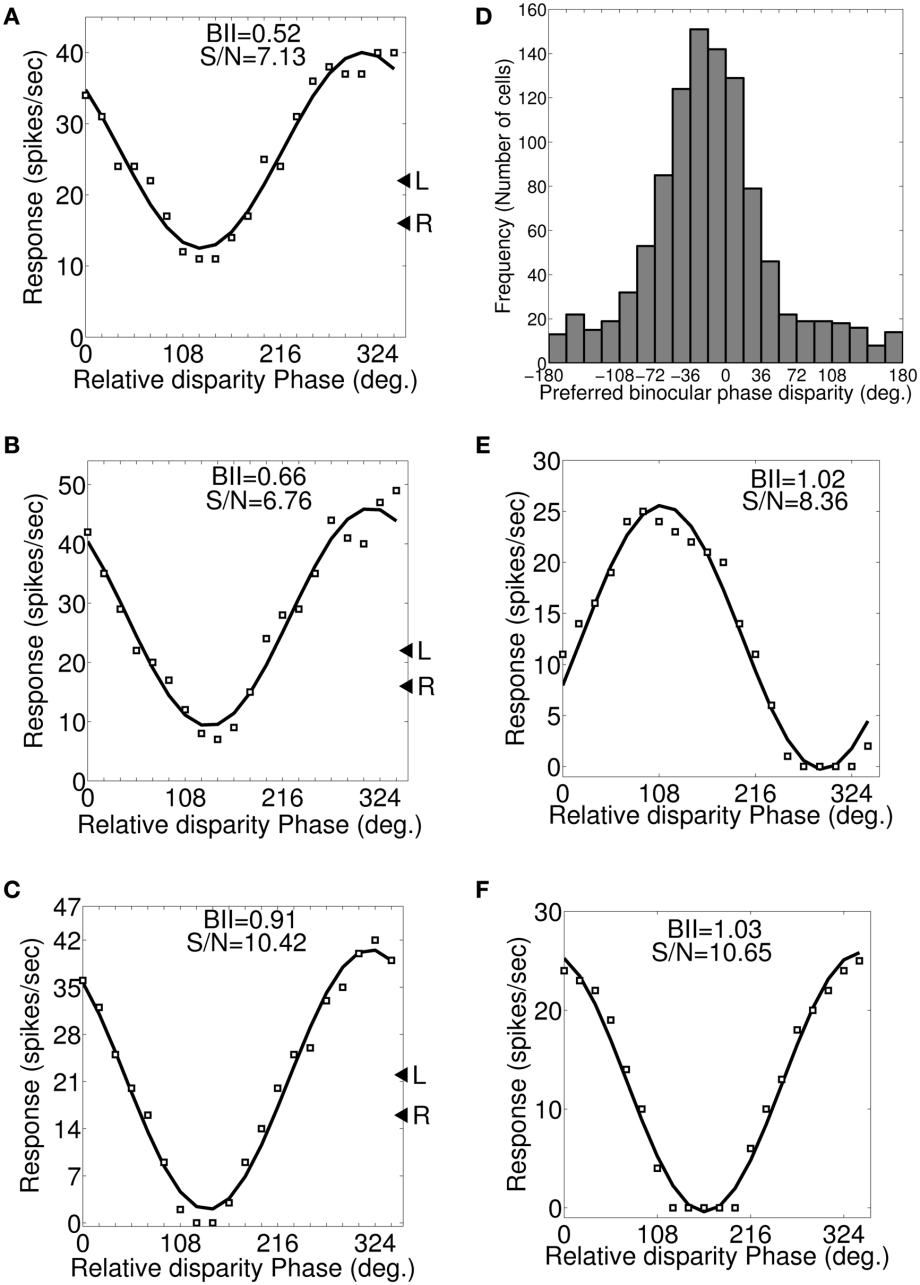
**Preferred binocular phase disparity for a dif-frequency cell. (A–C)** A set of three possible binocular phase disparity tuning for an exemplar cell having 0.7 and 0.5 cycles/degree preferred SFs in the left and the right eye. This set of binocular phase disparity tuning for the cell is obtained by dichoptically stimulating modeled retinae with drifting sinusoidal gratings at cell's preferred OR (72°) and SF at 0.5 cycles/degree (depicted in **A**), 0.6 cycles/degree (depicted in **B**) and 0.7 cycles/degree (depicted in **C**). The binocular phase disparity tuning depicted in **(C)** having left and right SF of 0.7 cycles/degree yields highest BII of 0.91. We, therefore, determined the preferred binocular phase disparity (DP) of the cell from the response characteristics shown in **(C)**. The DP for this cell is 318° (−42°) PA. **(D)** Histogram of preferred binocular phase disparity (DP) for dif-frequency selective cells in −180° to 180° scale. **(E,F)** Binocular phase disparity tuning curves for an exemplar cell with IDPO = 30° and matched inter-ocular SF preference.

Let *f*_*l*_ and *f*_*r*_ cycles/degree be the left and right eye preferred SFs in dif-frequency cells. We vary SF from *f*_*l*_ to *f*_*r*_ in steps of 0.1 cycles/degree. Figures 6A–C depicts a set of three binocular phase disparity tuning responses for a sample cell having 0.7 and 0.5 cycles/degree preferred SFs in the left and the right eye. The set of binocular phase disparity tuning response for the cell is obtained by dichoptically stimulating model retinae with drifting sinusoidal gratings at cell's preferred OR with SF at (1) 0.5 cycles/degree (Figure 6A), (2) 0.6 cycles/degree (Figure 6B), and (3) 0.7 cycles/degree (Figure 6C), respectively. The sample cell has matched orientation preferences in the left and the right eye. DP for this cell therefore is the same in the three plots shown in Figures 6A–C, only BII varies. DP is 318° (−42°) PA. For dif-frequency cells one can obtain DP either at *f*_*l*_ or *f*_*r*_ or at any frequency f lying between *f*_*l*_ and *f*_*r*_. The sample cell for Figure 6 detects fronto-parallel surface at a distance corresponding to DP = 318° PA. DP histogram for dif-frequency cells in the model cortex are shown in Figure 6D.

#### Cells with IDPO

Let θ_*l*_ and θ_*r*_ be the left and the right eye preferred orientations in cells with IDPO. Each cell is dichoptically stimulated with sinusoidal gratings at its preferred SF. The orientation of gratings applied to the left and the right eye are varied so as to get different combination of orientations. Let us consider a sample cell (see Table 3) with IDPO = 30° where θ_*l*_ = 88° and θ_*r*_ = 58°. The left and the right retina are dichoptically stimulated with drifting sinusoidal gratings at their respective preferred (Ohzawa and Freeman, 1986a) ORs and the corresponding binocular phase disparity response of the sample cell is shown in Figure 6E. DP of the cell is 111.3°. DP and BII values for the cell for different combinations of orientations of grating stimuli applied are listed in Table 3. For detecting fronto-parallel surface the orientation of the grating stimuli should be same for both the eyes. Figure 6F shows the response of the same cell when the grating applied to both the left and the right eye are kept at the preferred orientation for the left eye. Note that DP in this case is 343.1°. The best BII for the cell is obtained when grating orientations are the same as the preferred orientation of the right eye. The corresponding DP is 328.2° PA. The cell detects fronto-parallel surface at a distance corresponding to DP = 328.2° PA. We have obtained DP for all the cells with IDPO in our model cortex.

**Table 3:** **DP, BII, and S/N for a modeled cortical cell having unmatched OR and matched SF preference**.

**Preferred OR (degree): Left eye = 88, Right eye = 58**					
**IDPO (degree) = 30**					
**Preferred SF (cycles/degree): Left eye = Right eye = 0.5**					
**Dif-frequency (cycles/degree) = 0**					
OR (degree) of grating for left eye	90	90	72	54	90
OR (degree) of grating for right eye	54	72	72	54	90
DP (degree)	111.3	57.3	330.6	343.1	328.2
BII	1.02	0.99	1.00	1.03	1.05
S/N	8.36	7.57	8.46	10.65	10.47

The range of phase disparities of the cells in the model cortex lies within ±1.40 VA (*S* = 0.39). It is interesting to note that most binocular disparities in humans in natural surroundings fall within the range of ±1.50 VA (Geisler, 2008).

## Discussion

Slant about a vertical axis causes relative compression of a surface viewed by the two eyes and leads to a difference in spatial frequency content of the left and the right eye images. Neurons with inter-ocular spatial frequency differences (dif-frequency cells) are, therefore, expected to detect vertical surface slant. We have obtained RFs for cortical cells in layer IV of cat V1 and studied the sensitivity of dif-frequency cells in detecting the vertical surface slant. We now discuss whether our conclusion regarding lack of vertical slant selectivity would hold for dif-frequency cells in monkey V1.

**Figure 7:**
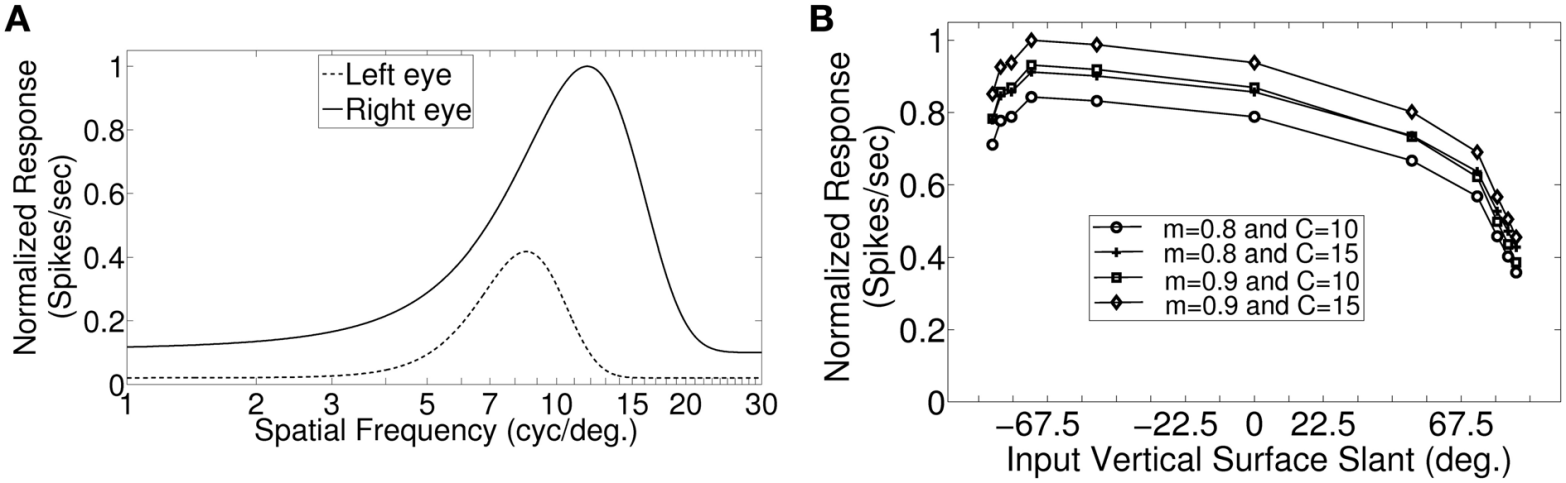
**Monocular SF tuning characteristics of a hypothetical monkey cell H and its vertical surface slant tuning. (A)** The left and the right eye SF tuning characteristics of a hypothetical monkey cell H. **(B)** Vertical slant tuning for cell H.

In macaque V1 the spatial frequency preferences for neurons range from 0.5 to 8.0 cycles/degree and spatial frequency bandwidth is 1.4 octaves (Foster et al., 1985). The difference in preferred spatial frequency between the two eyes ranges from small values to substantially large values of over an octave (Read and Cumming, 2003). Using a Gaussian function we have constructed the monocular spatial frequency response plot similar to the plot for a monkey neuron shown in Figure 7A in (Read and Cumming, 2003). Let us say that the responses shown in Figure 7A belong to a hypothetical monkey cell H. We now estimate the slant selectivity of cell H from its monocular response. Let L be the left monocular response to a grating of spatial frequency, *f*_*l*_, applied to the left eye, and R be the right monocular response to a grating of spatial frequency, *f*_*r*_, applied to the right eye. Let B be the binocular response when left eye and right eyes see gratings of spatial frequency *f*_*l*_ and *f*_*r*_, respectively. The inputs from the two eyes add sub-linearly in binocular spiking response (Zhao et al., 2013). We therefore estimate the binocular response, B of cell H by adding the monocular responses sub-linearly. For sub-linear addition we use *B* = *m*(*L* + *R*) + *C* where, 0 < *m* < 1 and *C* > 0. We show the slant tuning response for cell H for the two different values of *m* (= 0.9, 0.8) and *C* (= 10, 15) in Figure 7B. Cell H lacks sensitivity to a vertical slant. In the model cat V1 the preferred SF range is much smaller than the preferred SF range in monkey. But slant tuning characteristic depends on the ratio of the monocular preferred spatial frequency responses in the two eyes and not on the range. The spatial frequency bandwidth (1.5 octave), and the difference in preferred spatial frequency (in octaves) between the two eyes in our model cortex are similar to that reported in monkey. We therefore expect that dif-frequency cells in monkey V1 would lack vertical slant selectivity. Electrophysiological data are required to confirm our conjecture regarding vertical slant sensitivity in monkies.

The orientation bandwidth (hwhh) for left and right eye in cells with IDPO are significantly correlated (*r* = 0.2, *p* < 0.05) in the model cat V1. The mean binocular bandwidth is 39° with a standard deviation of 13°. The orientation tuning bandwidth in the model IDPO cells are too large to signal orientation disparity in a scene. In the monkey V1, hwhh for left and right eye are also significantly correlated (*r* = 0.33, *p* < 0.05) (Bridge and Cumming, 2001) but mean orientation bandwidth in neurons with IDPO is narrower. The binocular tuning width is still quite large (see Figure 7 in Bridge and Cumming, 2001) for cells with IDPO in monkeys to effectively encode horizontal slant.

The current study shows that in the model cortex for cat V1 both dif-frequency cells and cells with IDPO do not encode slant effectively. Earlier Nienborg et al. (2004) had studied slant selectivity in monkey V1 using a horizontally orientated sinusoidal grating in depth or “corrugation”. The depth corrugations place a time-varying slant over the RF i.e., the tilt around the horizontal axis varies over time. Nienborg et al. (2004) have reported that cells in monkey V1 lack selectivity for vertical disparity gradient. Our findings are consistent with the reports that neurons in monkey V1 lack slant selectivity (Bridge and Cumming, 2001; Nienborg et al., 2004). Human observers are also very bad at detecting horizontal disparity gradients for large extended surfaces (Rogers and Graham, 1983). Spatial stereo resolution in human visual system is poor and one of the factors that limit stereo resolution in human is the ability to detect disparity gradient (Banks et al., 2004).

### Conflict of interest statement

The authors declare that the research was conducted in the absence of any commercial or financial relationships that could be construed as a potential conflict of interest.
